# Welcome to *Molecular Brain*

**DOI:** 10.1186/1756-6606-1-1

**Published:** 2008-06-17

**Authors:** Lin Mei, Kei Cho, C Justin Lee, Xiao-Jiang Li, Min Zhuo, Bong-Kiun Kaang

**Affiliations:** 1 Institute of Molecular Medicine and Genetics, Medical College of Georgia, Augusta, GA 30912, USA; 2 Henry Wellcome Laboratories, Faculty of Medicine and Dentistry, University of Bristol, Bristol BS1 3NY, UK; 3 Center for Neural Science, Korea Institute of Science and Technology, Seoul 136-791, Korea; 4 Department of Human Genetics, Emory University School of Medicine, Atlanta, GA 30322, USA; 5 Department of Physiology, University of Toronto, Toronto M5S 1A8, Canada; 6 Department of Biological Sciences, College of Natural Sciences, Seoul National University, Seoul 151-747, Korea

## Abstract

We are delighted to announce the arrival of a brand new journal dedicated to the ever-expanding field of neuroscience. *Molecular Brain *is a peer-reviewed, open-access online journal that aims at publishing high quality articles as rapidly as possible. The journal will cover a broad spectrum of neuroscience ranging from molecular/cellular to behavioral/cognitive neuroscience and from basic to clinical research. *Molecular Brain *will publish not only research articles, but also methodology articles, editorials, reviews, and short reports. It will be a premier platform for neuroscientists to exchange their ideas with researchers from around the world to help improve our understanding of the molecular mechanisms of the brain and mind.

## 

Neuroscience, the study of the nervous system, is not an emerging field [1]. In fact, discoveries in neuroscience were honored three times by the Nobel Prize in Physiology or Medicine within the first 10 years of its inception in 1901. They include the work by Santiago Ramon y Cajal who established the neuron doctrine and laid the foundation of modern neuroscience [2]. In the 100 years since then, brain regions have been mapped to individual functions, and much has been learned about brain functions at cellular and molecular levels with the identification of neurotransmitters and their receptors, ion channels, and proteins that regulate neurotransmission. We have begun to understand how the brain is wired up during development, how neurons are connected with one another, and how their connections are modified by experience [3]. Nevertheless, due to the extremely complex structure and function, the brain remains to be an organ that we know least about how it works under physiological and pathological conditions. In the genomic era, many genes have been implicated in development and functions of neural cells [4]. However, there is a vast space to be filled between our knowledge of molecular and cellular systems and those of higher-level mental functions, and between our knowledge of susceptibility genes and mutations and causal mechanisms in psychiatric and neurological disorders [5]. In anticipation of rapid advances in neuroscience in the post-genomic era, we are pleased to announce the launch of *Molecular Brain*.

## Open-access

*Molecular Brain *is an open-access, peer-reviewed online journal [6]. Open access means that all articles are freely and permanently available online immediately upon publication [7]. The open-access publishing model ensures research published in the journal is highly visible and can be read by a global audience. In an era when online journals are emerging at an unprecedented speed, we believe that *Molecular Brain *will provide a valuable contribution to the rapidly expanding and diverse neuroscience field. As an online, open access journal, *Molecular Brain *will provide a home for high quality research on molecular, cellular and system levels of the physiological and pathological brain.

## Scope

The word "molecular" in the title of the journal is not meant to limit the journal's scope. The journal covers a broad spectrum of neuroscience and publishes studies of a wide range of topics in the field: from molecules to behavior, from basic science to clinical research, and from invertebrates to vertebrates, at the molecular, cellular, and systems levels. We especially encourage the submissions of works that contain studies at both molecular and behavioral levels. *Molecular Brain *publishes not only research articles, but also methodology articles, editorials, reviews on timely issues and short reports. It will provide a forum for neuroscientists to communicate news, thoughts, and ideas.

## Peer review

*Molecular Brain *aims to publish high quality articles as rapidly as possible, usually within 1 month. Our editors are committed to providing speedy reviews. We will constantly consider ways to improve the review process with one goal in mind: speed and quality. To ensure quality, *Molecular Brain *fully utilizes the journal's expert Editorial Board and reviewers from around the world in providing the most current perspective on a specific field of neuroscience. *Molecular Brain *operates a closed peer review process. Manuscripts will initially be evaluated by one of the Editors-in-Chief who will decide whether further peer review is warranted. An academic decision will be made based on the recommendations of at least two expert reviewers.

## Closing

As a top-quality open-access online journal, *Molecular Brain *aims to provide a home for the latest research from neuroscientists all over the world. Members of the Editorial Board have a broad range of expertise representing diverse areas of neuroscience. Importantly they are committed to the success of *Molecular Brain *by serving as reviewers and publishing their own important work in the journal. Our goal is to make *Molecular Brain *a premier journal in neuroscience within five years. This will not be possible without your support. We welcome your contributions and invite you to join us in creating this new and high quality platform that is *Molecular Brain*.

## Congratulatory remark on *Molecular Brain*

"It is a pleasure to extend a welcome to the first issue of *Molecular Brain*. The tools available for the exploration of the brain have now reached such a level of sophistication that, along with most neuroscientists, I believe this will be a golden age for research into the most complex structure in biology. The launch of *Molecular Brain *reflects this optimism, and with its strong editorial board and open-access policy it is well placed to play a leading role in the opening up of biology's last frontier." *-Tim V. P. Bliss (National Institute for Medical Research, UK*)
